# Comparison template-based with CT-based attenuation correction for hybrid MR/PET scanners

**DOI:** 10.1186/2197-7364-1-S1-A47

**Published:** 2014-07-29

**Authors:** Elena Rota Kops, Hans Herzog, N Jon Shah

**Affiliations:** Institute of Neuroscience and Medicine-4, Forschungszentrum Juelich, Kragujevac, Germany

Attenuation correction (AC) of PET data acquired in hybrid MR/PET scanners is still a challenge, even for brain imaging. A correction method previously proposed by our group and obtained with template-based attenuation maps (AM) used MR and ECAT HR+ images. In this study we investigated the influence of both template-based and CT-based AC methods on reconstructed images.

The data of two volunteers, measured in the Siemens 3T MR-BrainPET scanner, were used. Corresponding CT images were acquired. Due to the different resolution of ECAT and CT-derived AMs 3D Gaussian kernels with 3, 5, 7, and 9 mm filter width were applied to the latter ones. The CT-based AM with 3 mm filter was considered as reference. The BrainPET emission data were reconstructed with the five AMs: AMtemplate and AMCTxmm (with x = 3, 5, 7, and 9). The reconstructed PET images were normalized to the MNI brain for using the AAL-VOI Atlas analysis. The final brain regions considered were the frontal, temporal, parietal, occipital, and cerebellum, whith the corresponding AAL-VOIs merged to one single VOI. Furthermore, caudate nucleus, putamen, and thalamus were considered. The relative differences between AMCT3mm and all other four AMs were calculated.

Considering first only the CT-derived AMs the relative differences ranged between 0.11% (putamen area with AMCT9mm) and 2.70% (merged cerebellum area with AMCT9mm). The comparison of AMCT3mm with AMtemplate over all VOIs showed an overall, equally distributed underestimation of 8.0%.

The template-based AC method proposed by our group does not show any remarkable differences when compared to ECAT data (results not shown here). At the same time provides a reliable AC method for brain imaging measured in MR-PET scanners. Nevertheless, the number of tested subjects is still low and an analysis of a larger dataset is an ongoing running project.

